# Identification of Type VI Secretion Systems Effector Proteins That Contribute to Interbacterial Competition in *Salmonella* Dublin

**DOI:** 10.3389/fmicb.2022.811932

**Published:** 2022-02-10

**Authors:** Fernando A. Amaya, Carlos J. Blondel, María F. Barros-Infante, Dácil Rivera, Andrea I. Moreno-Switt, Carlos A. Santiviago, David Pezoa

**Affiliations:** ^1^Laboratorio de Microbiología, Departamento de Bioquímica y Biología Molecular, Facultad de Ciencias Químicas y Farmacéuticas, Universidad de Chile, Santiago, Chile; ^2^Instituto de Ciencias Biomédicas, Facultad de Medicina y Facultad de Ciencias de la Vida, Universidad Andrés Bello, Santiago, Chile; ^3^Escuela de Medicina Veterinaria, Facultad de Ciencias, Universidad Mayor, Santiago, Chile; ^4^Escuela de Medicina Veterinaria, Facultad de Ciencias de la Vida, Universidad Andrés Bello, Santiago, Chile; ^5^Escuela de Medicina Veterinaria, Facultad de Agronomía e Ingeniería Forestal, Facultad de Ciencias Biológicas y Facultad de Medicina, Pontificia Universidad Católica de Chile, Santiago, Chile; ^6^Millennium Initiative on Collaborative Research on Bacterial Resistance (MICROB-R), Santiago, Chile

**Keywords:** *Salmonella* Dublin, interbacterial competition, T6SS, effector, immunity protein

## Abstract

The Type VI Secretion System (T6SS) is a multiprotein device that has emerged as an important fitness and virulence factor for many Gram-negative bacteria through the injection of effector proteins into prokaryotic or eukaryotic cells *via* a contractile mechanism. While some effector proteins specifically target bacterial or eukaryotic cells, others can target both types of cells (trans-kingdom effectors). In *Salmonella*, five T6SS gene clusters have been identified within pathogenicity islands SPI-6, SPI-19, SPI-20, SPI-21, and SPI-22, which are differentially distributed among serotypes. *Salmonella enterica* serotype Dublin (*S*. Dublin) is a cattle-adapted pathogen that harbors both T6SS_SPI-6_ and T6SS_SPI-19_. Interestingly, while both systems have been linked to virulence and host colonization in *S*. Dublin, an antibacterial activity has not been detected for T6SS_SPI-6_ in this serotype. In addition, there is limited information regarding the repertoire of effector proteins encoded within T6SS_SPI-6_ and T6SS_SPI-19_ gene clusters in *S*. Dublin. In the present study, we demonstrate that T6SS_SPI-6_ and T6SS_SPI-19_ of *S*. Dublin CT_02021853 contribute to interbacterial competition. Bioinformatic and comparative genomic analyses allowed us to identify genes encoding three candidate antibacterial effectors located within SPI-6 and two candidate effectors located within SPI-19. Each antibacterial effector gene is located upstream of a gene encoding a hypothetic immunity protein, thus conforming an effector/immunity (E/I) module. Of note, the genes encoding these effectors and immunity proteins are widely distributed in *Salmonella* genomes, suggesting a relevant role in interbacterial competition and virulence. Finally, we demonstrate that E/I modules SED_RS01930/SED_RS01935 (encoded in SPI-6), SED_RS06235/SED_RS06230, and SED_RS06335/SED_RS06340 (both encoded in SPI-19) contribute to interbacterial competition in *S*. Dublin CT_02021853.

## Introduction

Salmonellosis is a foodborne bacterial disease caused by different serotypes of *Salmonella enterica* (GBD-2017 et al., 2019). Worldwide, this illness is linked to 95.1 million cases of gastroenteritis per year (GBD-2017 et al., 2019). The genus *Salmonella* includes more than 2,600 serotypes (also referred to as serovars) distributed between species *S. enterica* and *S. bongori* (Issenhuth-Jeanjean et al., 2014), which differ in clinical signs and host range (Uzzau et al., 2000). *Salmonella enterica* serotype Dublin (*S*. Dublin) represents a cattle-adapted pathogen that can lead to serious economic problems in bovine production, where it causes a severe systemic disease (Hoelzer et al., 2011; Nielsen et al., 2013). In addition, *S*. Dublin may constitute a serious risk for public health due to ingestion of contaminated milk by the human population (Small and Sharp, 1979; Fierer, 1983).

The Type VI Secretion System (T6SS) has emerged as an important fitness and virulence factor for many Gram-negative bacteria (Records, 2011; Basler, 2015; Alteri and Mobley, 2016; Cianfanelli et al., 2016; Navarro-Garcia et al., 2019). The T6SS is a molecular nanomachine consisting of three main complexes: a contractile tail, a membrane complex, and a baseplate (Aschtgen et al., 2008; Zoued et al., 2014; Durand et al., 2015; Brunet et al., 2015b; Logger et al., 2016; Nazarov et al., 2018; Rapisarda et al., 2019; Yin et al., 2019). The contractile tail is composed of an internal tube generated by the polymerization of a hexameric protein called Hcp, where a needle-shaped VgrG protein trimer is assembled at the tip. VgrG proteins are often associated with proteins harboring a N-terminal PAAR motif that sharpen the tip (Ballister et al., 2008; Shneider et al., 2013; Brunet et al., 2014, 2015b; Douzi et al., 2014; Zoued et al., 2014; Renault et al., 2018). The internal tube is surrounded by a contractile sheath formed by the polymerization of TssB and TssC subunits (Leiman et al., 2009; Basler, 2015; Durand et al., 2015). Contraction of the sheath provides the energy required for the injection of effector proteins that are confined within the Hcp tube, bound to VgrG and/or associated with PAAR proteins (Silverman et al., 2013). Secreted effectors are delivered fused to VgrG and/or PAAR proteins (evolved or specialized effectors) or through non-covalent interaction with some core components (cargo effectors) (Durand et al., 2014; Whitney et al., 2014; Diniz and Coulthurst, 2015; Ma et al., 2017b; Pissaridou et al., 2018). Notably, both antibacterial and antieukaryotic effector proteins have been identified (Feria and Valvano, 2020; Hernandez et al., 2020), highlighting the role of T6SSs as key players in processes, such as interbacterial competition and host–pathogen interaction (Ma and Mekalanos, 2010; Russell et al., 2012, 2013; Koskiniemi et al., 2013; Miyata et al., 2013; Srikannathasan et al., 2013; Whitney et al., 2013; Egan et al., 2015; Bondage et al., 2016; Flaugnatti et al., 2016; Tang et al., 2018; Ting et al., 2018; Ahmad et al., 2019; Berni et al., 2019; Coulthurst, 2019; Jana et al., 2019; Mariano et al., 2019; Wood et al., 2020). Antibacterial effectors include, among others, those targeting the peptide or glycosidic bonds of peptidoglycan (Ma and Mekalanos, 2010; Russell et al., 2012; Srikannathasan et al., 2013; Whitney et al., 2013; Berni et al., 2019; Wood et al., 2019), or the FtsZ cell division ring (Ting et al., 2018). These antibacterial effectors are usually encoded along with their cognate immunity proteins in bicistronic elements known as effector/immunity (E/I) modules. Immunity proteins bind tightly and specifically to their cognate effector preventing self-intoxication and killing of sibling cells (Russell et al., 2012). Antieukaryotic effectors include, among others, those targeting the actin or microtubule cytoskeleton networks, the endoplasmic reticulum, lipid membranes, and others that activate the AIM2 inflammasome and decrease the levels of reactive oxygen species contributing to survival in macrophages (Pukatzki et al., 2007; Ma et al., 2009; Ma and Mekalanos, 2010; Miyata et al., 2011; Zheng et al., 2011; Durand et al., 2012; Lindgren et al., 2013; Schwarz et al., 2014; Heisler et al., 2015; Sana et al., 2015; Aubert et al., 2016; Jiang et al., 2016; Ray et al., 2017; Dutta et al., 2019; Tan et al., 2019; Wood et al., 2019). In addition, effectors presenting both antibacterial and antieukaryotic activity (defined as trans-kingdom effectors) include, among others, those targeting conserved molecules (e.g., NAD) or macromolecules (e.g., DNA and phospholipids), or those forming pores in biological membranes (Whitney et al., 2015; Tang et al., 2018; Ahmad et al., 2019).

In *Salmonella*, five T6SS gene clusters have been identified within pathogenicity islands SPI-6, SPI-19, SPI-20, SPI-21, and SPI-22 (Blondel et al., 2009; Fookes et al., 2011). These T6SSs are distributed in four different evolutionary lineages: T6SS_SPI-6_ belongs to subtype i3, T6SS_SPI-19_ to subtype i1, T6SS_SPI-20_ and T6SS_SPI-21_ to subtype i2, and T6SS_SPI-22_ to subtype i4a (Bao et al., 2019). In addition to their distinct evolutionary origin, these five T6SS clusters are differentially distributed among distinct serotypes, subspecies, and species of *Salmonella* (Blondel et al., 2009; Fookes et al., 2011).

Notably, while both T6SS_SPI-6_ and T6SS_SPI-19_ have been linked to antibacterial competition, virulence, and host colonization in different *Salmonella* serotypes (Blondel et al., 2010, 2013; Libby et al., 2010; Wang et al., 2011, 2019; Mulder et al., 2012; Pezoa et al., 2013; Koskiniemi et al., 2014; Brunet et al., 2015a; Sana et al., 2016; Schroll et al., 2019; Sibinelli-Sousa et al., 2020; Xian et al., 2020), there is limited information regarding the effector proteins encoded within the corresponding gene clusters among different *Salmonella* serotypes. Furthermore, the contribution of both T6SSs to these phenotypes seems to differ between strains within the same serotype (Schroll et al., 2019). This appears to be the case for different *S*. Dublin strains. While T6SS_SPI-19_ has been shown to be dispensable for colonization of either chickens or mice by *S*. Dublin CT_02021853 (Pezoa et al., 2014), it has been shown to be required for efficient intestinal colonization of mice by *S*. Dublin 2229 (Schroll et al., 2019). Furthermore, it has been reported that T6SS_SPI-19_ of *S*. Dublin 2229 contributes to interbacterial competition (Schroll et al., 2019), reminiscent of what has been shown for T6SS_SPI-6_ of *S*. Typhimurium (Brunet et al., 2015a; Sana et al., 2016). Interestingly, the contribution of T6SS_SPI-6_ to interbacterial competition has not been assessed in *S*. Dublin and there is a lack of information regarding the presence of antibacterial and/or antieukaryotic effector proteins encoded in the SPI-6 and SPI-19 T6SS gene clusters present in this serotype.

In the present study, we evaluated the contribution of T6SS_SPI-6_ and T6SS_SPI-19_ to interbacterial competition by *S*. Dublin CT_02021853 and performed an *in silico* analysis of both T6SS gene clusters to identify putative effector and cognate immunity proteins. First, we observed that *S*. Dublin CT_02021853 outcompeted a susceptible *Escherichia coli* strain in the presence of bile salts, as reported in the case of *S*. Dublin 2229 (Schroll et al., 2019). In addition, we found that both T6SS_SPI-6_ and T6SS_SPI-19_ contribute to interbacterial competition by this strain. Subsequently, a comprehensive bioinformatic analysis identified five high-confidence potential new effector and immunity proteins encoded within SPI-6 and SPI-19 T6SS gene clusters in *S*. Dublin CT_02021853. The bioinformatic analysis was based on four distinct criteria, including prediction of E/I modules by the Bastion6 prediction pipeline, identification of conserved domains and motifs linked to known T6SS effectors, and remote homology prediction by the HHpred HMM-HMM prediction pipeline. Finally, we confirmed the participation of E/I modules SED_RS01930/SED_RS01935, SED_RS06235/SED_RS06230, and SED_RS06335/SED_RS06340 of *S*. Dublin CT_02021853 in interbacterial competition.

## Materials and Methods

### Bacterial Strains and Growth Conditions

The bacterial strains used in this study are listed in Table 1. Bacteria were routinely grown in Luria-Bertani (LB) broth (10 g/L tryptone, 5 g/L yeast extract, 5 g/L NaCl) at 37°C with aeration. LB broth was supplemented with ampicillin (Amp; 100 μg/ml), kanamycin (Kan; 50 μg/ml), chloramphenicol (Cam; 20 μg/ml), or nalidixic acid (Nal; 15 μg/ml), as needed. LB medium was solidified by addition of agar (15 g/L). For interbacterial competition assays bacteria were incubated on MacConkey agar plates at 37°C for 24 h.

**Table 1:** Bacterial strains and plasmids used in this study.

Strains	Features	Source or reference
**Escherichia coli**		
DH5α	F^−^ Φ80∆*lacZ*(M15) ∆(*lacZYA-argF*)*U169* deoR *recA1 endA1 hsdR17*(r_k_^−^, m_k_^+^) *phoA supE44 thi-1 gyrA96 relA1* λ^−^	Laboratory collection
**Salmonella Dublin**		
CT_02021853	Wild-type strain	Laboratory collection
ΔT6SS_SPI-6_	CT_02021853 Δ(SED_RS01790-SED_RS25725)::Kan	This study
ΔT6SS_SPI-19_	CT_02021853 Δ(SED_RS06220-SED_RS06380)::Kan	This study
ΔT6SS_SPI-6_ ΔT6SS_SPI-19_	CT_02021853 Δ(SED_RS01790-SED_RS25725)::FRT Δ(SED_RS06220-SED_RS06380)::Cam	This study
Δ*phoN*::Kan	CT_02021853 Δ(SED_RS22655)::Kan	This study
Δ(SED_RS01930)::FRT Δ*phoN*::Kan	CT_02021853 Δ(SED_RS01930)::FRT Δ(SED_RS22655)::Kan	This study
Δ(SED_RS01930)::FRT Δ*phoN*::Cam	CT_02021853 Δ(SED_RS01930)::FRT Δ(SED_RS22655)::Cam	This study
Δ(SED_RS01930-SED_RS01935)::FRT Δ*phoN*::Cam	CT_02021853 Δ(SED_RS01930-SED_RS01935)::FRT Δ(SED_RS22655)::Cam	This study
Δ(SED_RS06235)::FRT Δ*phoN*::Kan	CT_02021853 Δ(SED_RS06235)::FRT Δ(SED_RS22655)::Kan	This study
Δ(SED_RS06235)::FRT Δ*phoN*::Cam	CT_02021853 Δ(SED_RS06235)::FRT Δ(SED_RS22655)::Cam	This study
Δ(SED_RS06235-SED_RS06230)::FRT Δ*phoN*::Cam	CT_02021853 Δ(SED_RS06235-SED_RS06230)::FRT Δ(SED_RS22655)::Cam	This study
Δ(SED_RS06335)::FRT Δ*phoN*::Kan	CT_02021853 Δ(SED_RS06335)::FRT Δ(SED_RS22655)::Kan	This study
Δ(SED_RS06335)::FRT Δ*phoN*::Cam	CT_02021853 Δ(SED_RS06335)::FRT Δ(SED_RS22655)::Cam	This study
Δ(SED_RS06335-SED_RS06340)::FRT Δ*phoN*::Cam	CT_02021853 Δ(SED_RS06335-SED_RS06340)::FRT Δ(SED_RS22655)::Cam	This study
**Plasmids**		
pKD46	*bla P_BAD_ bet gam exo oriR101*(TS), Amp^R^	Datsenko and Wanner, 2000
pCP20	*bla cat cI857* λP_R_ *flp oriR101*(TS), Cam^R^, Amp^R^	Cherepanov and Wackernagel, 1995
pCLF2	Red-swap redesigned vector, Cam^R^	GenBank HM047089
pCLF4	Red-swap redesigned vector, Kan^R^	GenBank EU629214

### Standard DNA Techniques

Plasmid DNA was isolated using the “QIAprep Spin Miniprep Kit” (QIAGEN, MD, United States). PCR products were purified using the “QIAquick PCR Purification Kit” (QIAGEN, MD, United States). DNA samples were analyzed by electrophoresis in 1% agarose gels and were visualized under UV light after RedGel (Biotium, CA, United States) staining. Primers were designed using the “Vector NTI Advance 11.0” software (Invitrogen, CA, United States) and are listed in Table 2. PCR products were amplified in a “MultiGene TC9600-G” thermal cycler (LabNet, NJ, United States). PCR reaction mixes contained 1X buffer, 2 mM MgCl_2_, 100 nM dNTPs, 100 nM of each primer, 100 ng of template DNA and 0.5–1 U of HiFi DNA pol (KAPA, MA, United States). Standard conditions for amplification were: 2 min at 95°C, followed by 30–35 cycles of 94°C for 45 s, 55°C for 30 s, and 72°C for a suitable time (1 min/kb) according to DNA polymerase processivity, and a final extension step at 72°C for 5 min.

**Table 2:** Primers used in this study.

Primer	Sequence^a^
SPI-6_T6SS_(H1 + P1)	AGGGTGTTTTTATACATCCTGTGAAGTAAAAAAAACCGTA*GTGTAGGCTGGAGCTGCTTC*
SPI-6_T6SS_(H2 + P2)	GTGAACATGGCACATTAATTTGAAGCAGCTCTCATCCGGT*CATATGAATATCCTCCTTAG*
SPI-6_T6SS_Out5	CCGAAGTGTATCTGGCGATGA
SPI-19_T6SS_(H1 + P1)	TAGCTGAATTGCAATATGCGAAAAAAGCCGAGCTTGATGACAAAC*GTGTAGGCTGGAGCTGCTTC*
SPI-19_T6SS_(H2 + P2)	AAGCATCTTCAATAATCACGGGTATAAATGCTTACACTCTTTATC*CATATGAATATCCTCCTTAG*
SPI-19_T6SS_Out5	ATCCGGCATGTTCTTGCG
SED_RS01930_(H1 + P1)	CAACGACTGCATGACGATGCACCGGGAGCCGGGCGGCGAA*GTGCAGGCTGGAGCTGCTTC*
SED_RS01930_(H2 + P2)	TCATCAAGAGTCATGATATTGGCCTTTGAGGTTTGGATGG*CATATGAATATCCTCCTTAG*
SED_RS01935_(H2 + P2)	CCGGCTGTCATTATATCTTATCTGATACTGAAAAACCAAA*CATATGAATATCCTCCTTAG*
SED_RS01930_Out5	ACCTTCAATACAGCCCCACA
SED_RS06235_(H1 + P1)	AGGGTTGCACATGGTAAATCGCACAGCATCGGCACACAAA*GTGCAGGCTGGAGCTGCTTC*
SED_RS06235_(H2 + P2)	CTTGTAAACGTTATTTACTCTCATCTGCGACAATGAGAGC*CATATGAATATCCTCCTTAG*
SED_RS06230_(H2 + P2)	ATAATAACCTCTATATATAATCGTTAAGCCATTTTATTTG*CATATGAATATCCTCCTTAG*
SED_RS06235_Out5	TTTCTCGATTGCGCATGTAGTC
SED_RS06335_(H1 + P1)	AGAAATAAAGATGAGCGGAAAACCAGCGGCGCGTCAGGGC*GTGCAGGCTGGAGCTGCTTC*
SED_RS06335_(H2 + P2)	ATCTTTATCATCAGTATTTCATCCTTGGTGGGATTCCCAT*CATATGAATATCCTCCTTAG*
SED_RS06340_(H2 + P2)	CTATGAAATATTAGTGATTATCTTCATATATATATATTCT*CATATGAATATCCTCCTTAG*
SED_RS06335_Out5	GCGGTATTTTTCTGAACGGCA
phoN_SDu_(H1 + P1)	GTGAGTCTTTATGAAAAGTCGTTATTTAGTATTTTTTCTA*GTGCAGGCTGGAGCTGCTTC*
phoN_SDu_(H2 + P2)	ACTTTCACCTTCAGTAATTAAGTTCGGGGTGATCTTCTTT*CATATGAATATCCTCCTTAG*
phoN_SDu_Out5	TTGCCTGATCCGGAGTGA
K1	CAGTCATAGCCGAATAGCCT
C3	CAGCTGAACGGTCTGGTTATAGG

a *Italics indicate the region that anneals to the 5′ or 3′ end of the antibiotic resistance cassette used for the mutagenesis*.

### Construction of *Salmonella* Dublin Mutant Strains

Derivatives of *S*. Dublin CT_02021853 with deletions of single genes or gene clusters were constructed by the one-step inactivation procedure using the Lambda Red recombination system (Datsenko and Wanner, 2000), with modifications (Santiviago et al., 2009). The oligonucleotides used for mutagenesis (Table 2) were designed with 40 bases at the 5′ ends identical to the ends of the corresponding deletion, and 20 bases at the 3′ ends that anneal with the 5′ or 3′ end of a Cam or Kan resistance cassette flanked by Flp recombinase target (FRT) sites present in plasmids pCLF2 (GenBank accession number HM047089) and pCLF4 (GenBank accession number EU629214.1), respectively. These plasmids were used as templates for the corresponding amplification of PCR products. *S*. Dublin CT_02021853 carrying the plasmid pKD46, which expresses the Lambda Red recombination system, was grown to an OD_600nm_ of 0.6 at 30°C in LB broth supplemented with Amp and L-arabinose (10 mM). Then, bacteria were made electrocompetent by serial washes with ice-cold, sterile 15% glycerol and transformed by electroporation with 500–600 ng of each PCR product. Transformants were selected on LB agar supplemented with the corresponding antibiotic at 37°C. Correct allelic replacement in each mutant was confirmed by PCR amplification using specific forward primers (Out5) together with reverse primer K1 (that hybridizes within the Kan resistance cassette) or reverse primer C3 (that hybridizes within the Cam resistance cassette; Table 2).

When required, the antibiotic resistance cassette was removed by transforming each mutant with the temperature-sensitive plasmid pCP20, which encodes the Flp recombinase (Cherepanov and Wackernagel, 1995). Transformants were selected at 30°C on LB agar plates containing Amp. A few colonies were streaked two consecutive times at 37°C on LB agar plates and tested for the loss of the antibiotic resistance cassettes and pCP20 by patching them on LB agar plates containing Kan or Cam plus Amp. The absence of the antibiotic resistance cassette was confirmed by PCR amplification using primers flanking the sites of substitution (Table 2). Finally, to differentiate between *S*. Dublin strains in our interbacterial competition assays, a Kan or Cam resistance cassette was incorporated at a neutral position (i.e., replacing the ORF of *phoN* gene) in the chromosome of the attacker or the prey strain, respectively. To do this, phage P22 HT 105-1 *int*-201 was used to transduce mutant alleles Δ*phoN*::Kan and Δ*phoN*::Cam into the corresponding mutant strains harboring unmarked deletions of genes encoding effectors or E/I protein pairs.

### Interbacterial Competition Assays

Competition assays were performed as described (Ma et al., 2018), with modifications. Briefly, attacker and prey bacteria were grown overnight in LB broth at 37°C. An aliquot (1 ml) of each culture was spun down, and the supernatant was discarded. Each bacterial pellet was washed three times in PBS, adjusted to an OD_600nm_ of 0.5, and mixed at a 1:1 (attacker/prey) ratio. Then, 25 μl of the mixture was incubated at 37°C for 24 h in triplicate on MacConkey agar plates, a condition reported to induce the expression of T6SS gene clusters in *Salmonella* (Schroll et al., 2019). After incubation, the bacterial mixtures were scraped from the plates and resuspended in 1 ml of PBS, and CFU were determined by plating of serial dilutions on LB agar supplemented with suitable antibiotics. Statistical significance was determined using a one-way ANOVA followed by Tukey’s multiple comparisons test using GraphPad Prism 9.0 software.

### Bioinformatics Analyses

To identify putative T6SS effectors encoded within SPI-6 and SPI-19 in *S*. Dublin CT_02021853, each ORF of both pathogenicity islands was analyzed with the Bastion6 pipeline (Wang et al., 2018) excluding those encoding the 13 structural components of the T6SS. ORFs presenting a Bastion6 score ≥ 0.7 were considered as putative T6SS effectors. Each Bastion6 prediction was also analyzed to determine if it was part of a bicistron encoding a putative immunity protein [i.e., a small protein with potential signal peptides (SignalP) and/or transmembrane domains (TMHMM)] with the Operon-Mapper web server (Taboada et al., 2018). Identification of conserved functional domains and motifs was performed using the PROSITE, NCBI-CDD, Motif-finder, and Pfam databases (Kanehisa et al., 2002; Sigrist et al., 2013; Finn et al., 2014; Lu et al., 2019) with the GenomeNet search engine. An e-value cutoff score of 0.01 was used. Finally, for each putative effector and immunity protein identified, a biochemical functional prediction was performed *via* HMM homology searches using the HHpred HMM-HMM comparison tool (Zimmermann et al., 2017).

### Sequence and Phylogenetic Analysis

Identification of T6SS effector orthologs was carried out using the DNA sequence of each effector/immunity module in BLASTn analyses using publicly available bacterial genome sequences of the NCBI database (January 2021). An 80% identity and 80% sequence coverage threshold were used to select positive matches. Sequence conservation was analyzed by multiple sequence alignments using MAFFT (Katoh et al., 2017) and T-Coffee Expresso (Notredame et al., 2000) and visualized by ESPript 3 (Robert and Gouet, 2014). Comparative genomic analysis of T6SS gene clusters was performed using the multiple aligner Mauve (Darling et al., 2004) and EasyFig v2.2.2 (Sullivan et al., 2011). Nucleotide sequences were analyzed by the sequence visualization and annotation tool Artemis version 18 (Rutherford et al., 2000).

## Results and Discussion

### The T6SSs Encoded in SPI-6 and SPI-19 Contribute to Interbacterial Competition by *S*. Dublin CT_02021853

**Figure 1:**
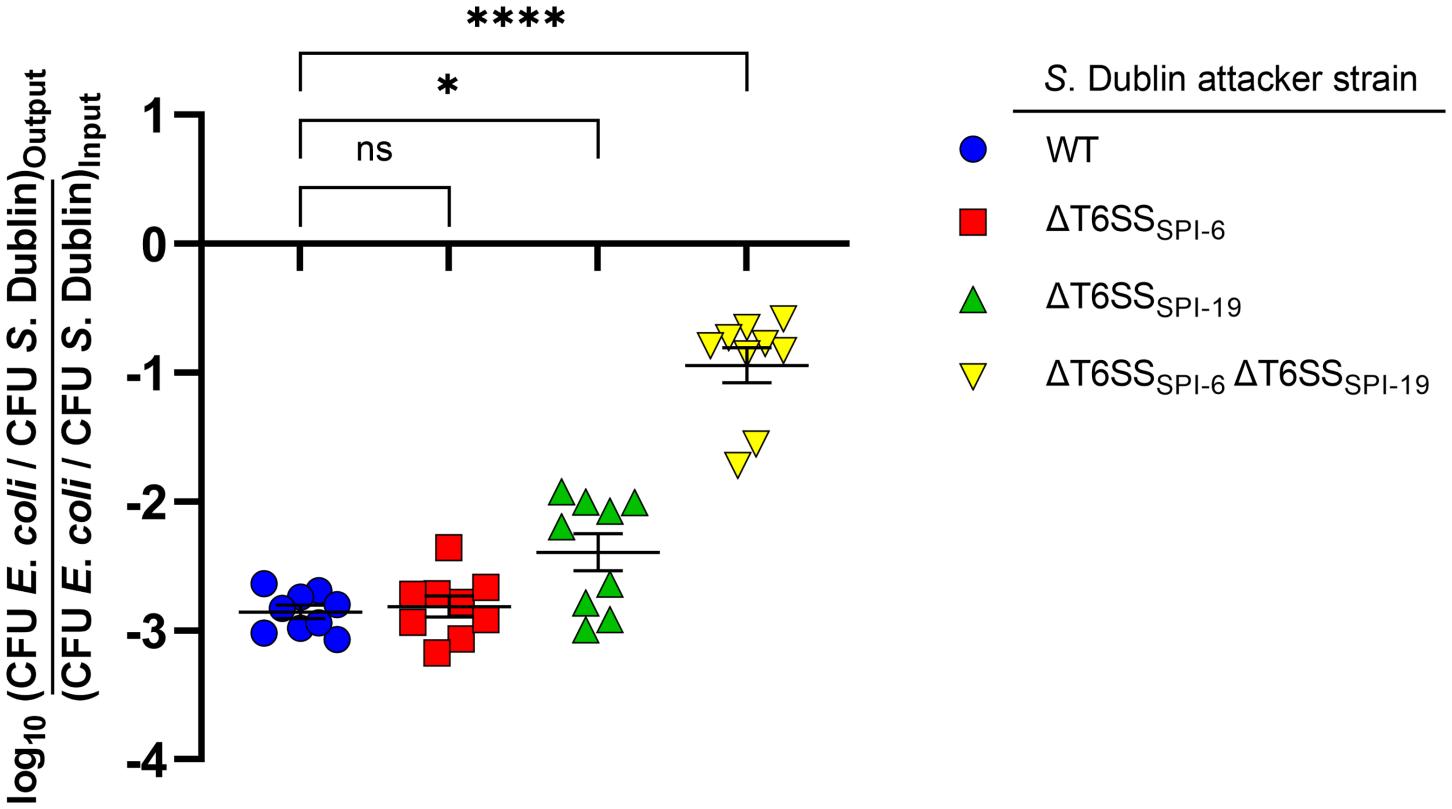
Contribution of T6SS_SPI-6_ and T6SS_SPI-19_ to interbacterial competition by *S*. Dublin strain CT_02021853. Wild-type and mutant strains ΔT6SS_SPI-6_, ΔT6SS_SPI-19_, and ΔT6SS_SPI-6_ ΔT6SS_SPI-19_ of *S*. Dublin CT_02021853 were mixed at a ratio of 1:1 (attacker/prey) with *Escherichia coli* DH5α. Then, 25 μl of the mixture was incubated at 37°C for 24 h in triplicate on MacConkey agar plates. Bacterial counts recovered from each competition assay were calculated by plating serial 10-fold dilutions on LB agar plates with the appropriate antibiotics (Nal in the case of *E. coli* and Kan or Cam in the case of *S*. Dublin strains). Data show the ratio of *E. coli* CFU (prey) to *S*. Dublin CFU (attacker) normalized to the inoculum ratio and expressed as log_10_. Error bars indicate standard error. Statistical significance was determined using a one-way ANOVA followed by Tukey’s multiple comparisons test (^*^p < 0.05; ^****^p < 0.0001; ns, not significant).

To determine the contribution of T6SS_SPI-6_ and T6SS_SPI-19_ to interbacterial competition by *S*. Dublin CT_02021853, we performed a competition assay in MacConkey agar as it has been shown that bile salts upregulate the expression of T6SS-related genes in *S*. Typhimurium and *S*. Dublin strains (Brunet et al., 2015a; Sana et al., 2016; Schroll et al., 2019). We decided to use mutant strains lacking the complete SPI-6 and/or SPI-19 T6SS gene clusters rather than single mutants in key structural components to avoid potential cross-complementation between components of both T6SS gene clusters as reported in other systems (Santos et al., 2020). We observed that the *E. coli* DH5α prey strain was significantly outcompeted after coincubation with *S*. Dublin CT_02021853. Of note, the ability of derivative strains ΔT6SS_SPI-6_ and ΔT6SS_SPI-19_ to outcompete the *E. coli* prey strain was similar to the wild-type strain, recovering 1,000-fold lower CFU of *E. coli* after coincubation (Figure 1). Remarkably, the ability of a ΔT6SS_SPI-6_ ΔT6SS_SPI-19_ double mutant to outcompete the *E. coli* prey strain was 100-fold lower than the wild-type strain and derivative strains ΔT6SS_SPI-6_ and ΔT6SS_SPI-19_ (Figure 1). These results indicate that T6SS_SPI-6_ and T6SS_SPI-19_ provide a competitive advantage to *S*. Dublin CT_02021853 over *E. coli* DH5α. Furthermore, having both T6SS systems does not provide an additive advantage over having only one, revealing a functional redundancy between both systems.

### The SPI-6 of *S*. Dublin CT_02021853 Encodes 3 Novel Putative T6SS Effector Proteins

**Figure 2:**
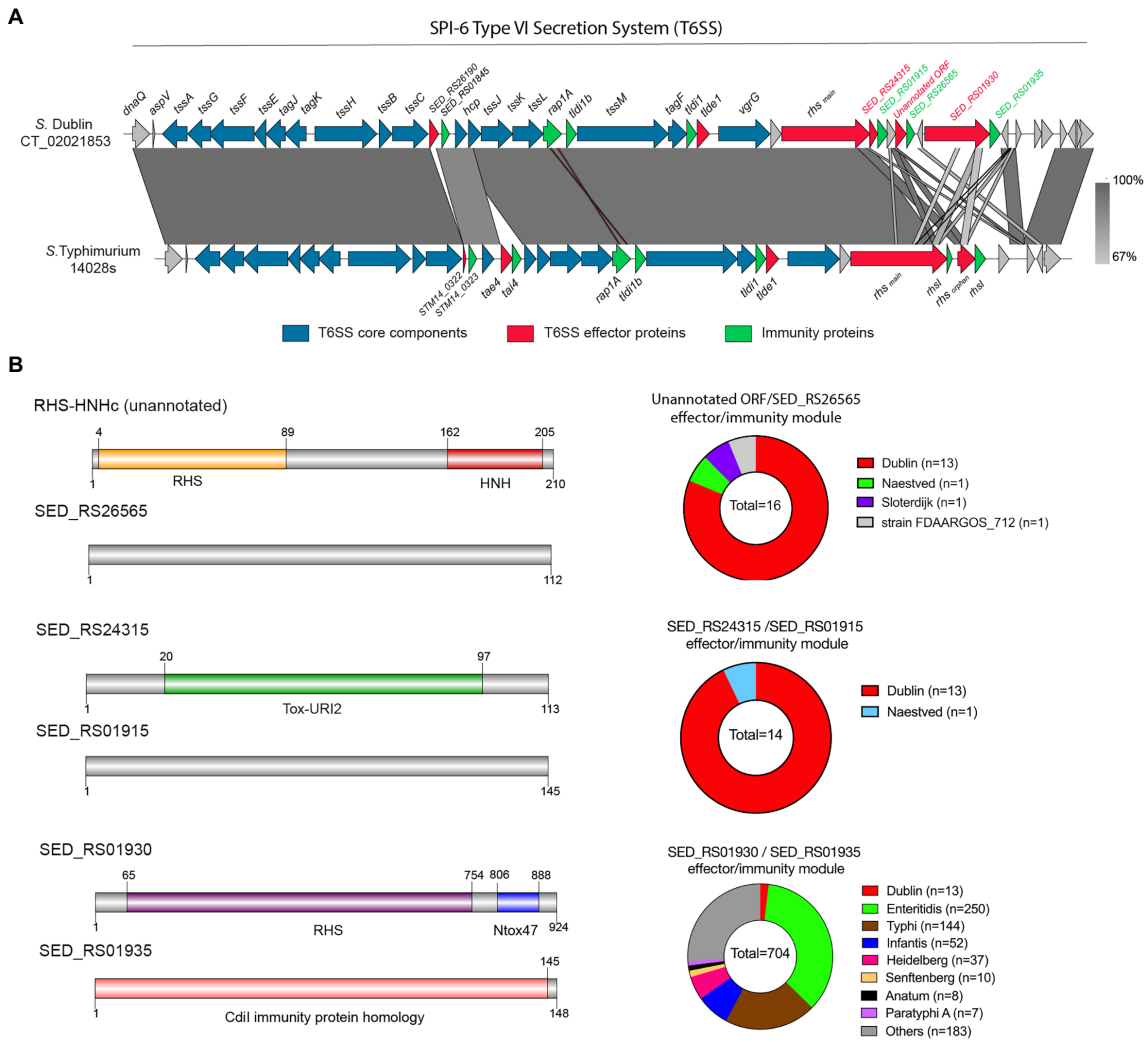
The SPI-6 T6SS gene cluster encodes novel putative Type VI Secretion Systems (T6SS) effector proteins. **(A)** Comparative genomic analysis of the SPI-6 T6SS cluster of *S*. Dublin CT_02021853 and *S*. Typhimurium 14028s. BLASTn sequence alignment was performed and visualized using EasyFig (Sullivan et al., 2011). **(B)** Schematic representation and distribution among *Salmonella* genomes of each novel effector and immunity protein identified. Names of genes encoding novel effectors and immunity proteins are highlighted in red and green, respectively. Homologs for each component were identified by BLASTn analyses as described in Materials and Methods.

*S*. Dublin CT_02021853 encodes 3 out of 4 antibacterial E/I pairs, as well as the two orphan immunity proteins, previously described in the SPI-6 T6SS gene cluster of *S. enterica* (Figure 2A): (i) Tae2/TaeI2 E/I pair (SED_RS26190/SED_RS01845) present in different *Salmonella* serotypes (Russell et al., 2012), (ii) the RHS_main_ (SED_RS01910) effector protein (Koskiniemi et al., 2014), and (iii) the Tlde1/Tldi1 E/I pair (SED_RS01895/SED_RS01890; Sibinelli-Sousa et al., 2020). In contrast, *S*. Dublin CT_02021853 does not encode the Tae4/Tai4 and RHS_orphan_/RhsI E/I pairs described in *S*. Typhimurium 14028s (Blondel et al., 2009; Koskiniemi et al., 2014; Figure 2A).

To gain insight into the T6SS-dependent antibacterial activity of *S*. Dublin, we performed bioinformatic and comparative genomic analyses to identify potential novel T6SS effector proteins and their cognate immunity proteins. To do this, each ORF encoded within the SPI-6 T6SS gene cluster of *S*. Dublin CT_02021853 was analyzed based on four criteria, including: (i) analysis by the Bastion6 prediction pipeline (a bioinformatics tool that predicts T6SS effectors based on amino acids sequence profile, evolutionary information, and physicochemical properties); (ii) bioinformatic analysis to identify the presence of putative immunity proteins through detection of signal peptide (SignalP), transmembrane domains (TMHMM), and operon prediction (Operon-mapper; Taboada et al., 2018); (iii) identification of conserved domains and motifs linked to known T6SS effectors (PROSITE, NCBI-CDD, Motif-finder, and Pfam databases); and (iv) functional predictions *via* HMM homology searches using the HHpred HMM-HMM prediction pipeline (Zimmermann et al., 2017). In addition, we analyzed the SPI-6 T6SS gene cluster to identify potential unannotated ORFs which could encode putative effectors and cognate immunity proteins. Our analysis identified three potential E/I pairs encoded within this T6SS gene cluster of *S*. Dublin CT_02021853 (Figure 2B; Table 3).

**Table 3:** Novel predicted T6SS effectors and cognate immunity proteins encoded in SPI-6 of *Salmonella* Dublin CT_02021853.

T6SS effector genes					Cognate T6SS immunity protein genes	
ORF (old locus annotation)	Size (aa)	Bastion6 T6SE (Score)	Target cell	Predicted activity/Domain	ORF/Upstream or downstream^a^	TM or signal peptide/Domain^b^
Unannotated ORF	210	0,873	Prokaryotic	DNase/RHS-HNH	SED_RS26565/Downstream	No/No
SED_RS01930(SeD_A0317)	924	0,909	Prokaryotic	RNase/RHS-Ntox47	SED_RS01935/Downstream	No/Cdi immunity protein homolog
SED_RS24315	113	0,733	Prokaryotic	DNase/Tox-URI2	SED_RS01915/Downstream	No/No

a This column indicates if the putative immunity protein gene (ORF) is encoded upstream or downstream the corresponding T6SS effector in a bicistronic unit.

b Presence or absence of transmembrane domains (TM) or a signal peptide and protein domains present in the putative immunity protein genes.

First, we identified an unannotated ORF located between the SED_RS24320 and SED_RS24325 ORFs. This novel ORF (Bastion6 score = 0,873) encodes a 210 amino acid protein with a predicted endonuclease RHS-HNHc protein domain (Figure 2B; Table 3). The presence of the HNHc endonuclease domain suggests that this putative protein harbors DNase activity, as described for other antibacterial and antieukaryote T6SS effector proteins (Ma et al., 2017a,b). SED_RS24325 is predicted to be co-transcribed with the downstream ORF SED_RS26565, suggesting that this latter ORF is the cognate immunity protein of the putative novel effector.

A second putative effector protein was predicted to be encoded by SED_RS1930. This ORF (Bastion6 score = 0,909) encodes a large 924 amino acid protein with both a RHS and an Ntox47 protein domain, involved in degrading target cell RNA (Zhang et al., 2012). It should be noted that the Ntox47 protein domain of SED_RS1930 has similarity to the Ntox47 domain of RHS_orphan_ protein of *S*. Typhimurium 14028s (Figure 2B). Since RHS orphan proteins have been linked to recombination events between RHS elements generating RHS_main-orphan_ chimeras (Koskiniemi et al., 2014), it is plausible that SED_RS1930 was generated through one of such events.

Finally, a third effector protein was predicted to be encoded in SED_RS24315. This ORF (Bastion6 score = 0,733) encodes a small 113 amino acid protein with similarity to bacterial polymorphic toxins in the Tox-URI2 family, a DNAse protein domain that has been linked to RHS-CT proteins exported by the T6SS (Zhang et al., 2012; Jamet and Nassif, 2015; Ma et al., 2017b). SED_RS24315 is predicted to be co-transcribed with SED_RS01915, suggesting that this latter ORF encodes the cognate immunity protein of the putative effector. Interestingly, the ORF just upstream of SED_RS24315 encodes the RHS_main_ protein (SED_RS01910). Taking into account that there are RHS proteins with C-terminal Tox-URI2 domains (Zhang et al., 2012; Ma et al., 2017b), it is possible that SED_RS01910 and SED_RS24315 were at some point a single ORF that was later split due to the accumulation of nonsense mutations.

Many T6SS-associated RHS proteins have C-terminal endonuclease effector domains, which degrade DNA or RNA in the target cell (Zhang et al., 2012). Interestingly, RHS proteins have YD-peptide repeats, which fold into a large β-cage structure that encapsulates and protects the C-terminal toxin domain and highly increase T6SS secretion efficiency (Donato et al., 2020), which could explain why many T6SS effectors are associated with RHS elements.

### The SPI-19 of *S*. Dublin CT_02021853 Encodes 2 Putative T6SS Effector Proteins

As mentioned, no effector protein has been described to be encoded within the SPI-19 T6SS gene cluster in *Salmonella*, despite its clear contribution to intestinal colonization and antibacterial activity (Blondel et al., 2010; Pezoa et al., 2014; Schroll et al., 2019; Xian et al., 2020). To identify novel T6SS effector proteins encoded within SPI-19, we performed bioinformatic analyses of each ORF included in this T6SS gene cluster of *S*. Dublin CT_02021853. In addition, we performed a comparative analysis of this T6SS gene cluster with the SPI-19 T6SS gene cluster of *S*. Gallinarum SG9, as T6SS_SPI-19_ activity in this strain causes cytotoxicity to primary macrophages from hens (Schroll et al., 2019).

**Figure 3:**
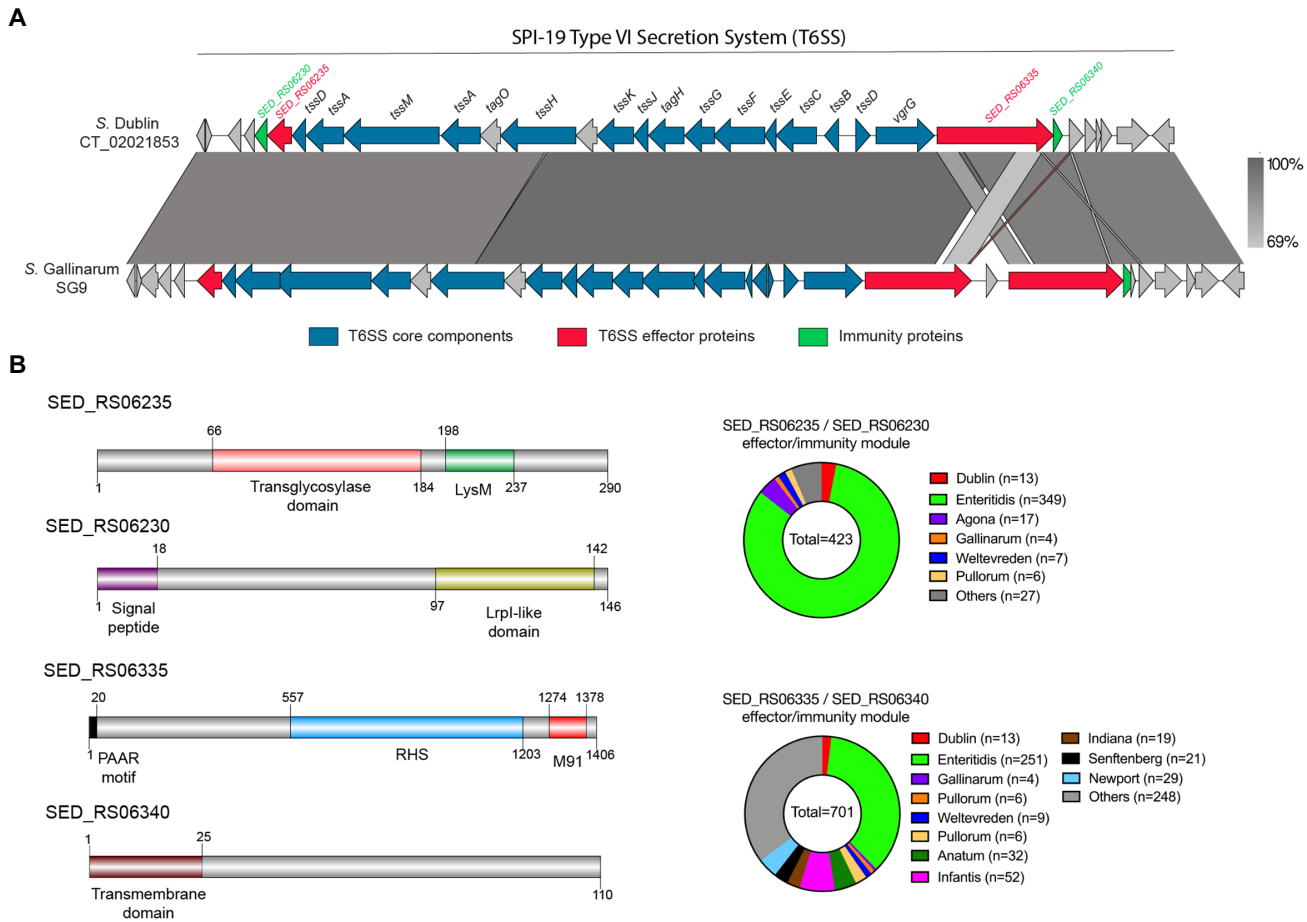
The SPI-19 T6SS gene cluster encodes novel putative T6SS effector proteins. **(A)** Comparative genomic analysis of the SPI-19 T6SS cluster of *S*. Dublin CT_02021853 and *S*. Gallinarum SG9. BLASTn sequence alignment was performed and visualized using EasyFig (Sullivan et al., 2011). **(B)** Schematic representation and distribution among *Salmonella* genomes of each novel effector and immunity protein identified. Names of genes encoding novel effectors and immunity proteins are highlighted in red and green, respectively. Homologs for each component were identified by BLASTn analyses as described in Materials and Methods.

Our analysis identified two putative T6SS effector proteins encoded in SPI-19 (Figure 3; Table 4), that are conserved in *S*. Gallinarum SG9 (Figure 3A). The first putative effector corresponds to SED_RS06335 (Bastion6 score = 0,939), an RHS protein that harbors an N-terminal PAAR domain and a C-terminal M91 metallopeptidase domain (Figure 3B). Our analysis showed that SED_RS06335 is part of a bicistronic unit with SED_RS06340. This latter ORF encodes a 110 amino acid protein with a transmembrane domain that may correspond to the cognate immunity protein of SED_RS06335. A comparative analysis with the SPI-19 T6SS gene cluster of *S*. Gallinarum SG9 revealed the presence of two copies of genes encoding this PAAR-RHS effector protein in this strain (SG9_1071 and SG9_1075; Figure 3A). Interestingly, a M91 metallopeptidase domain is present in the trans-kingdom effector VgrG2b of *Pseudomonas aeruginosa* (Wood et al., 2019). This M91 domain of VgrG2b has been shown to be involved in the internalization of *P. aeruginosa* into host cells by interacting with the host gamma-tubulin ring complex (Sana et al., 2015) and in the antibacterial activity of the H2-T6SS (Wood et al., 2019). Our analysis of SED_RS06335 showed the presence of the HEXXH motif typically found in the catalytic site of metallopeptidases (Supplementary Figure S1).

**Table 4:** Novel predicted T6SS effectors and cognate immunity proteins encoded in SPI-19 of *Salmonella* Dublin CT_02021853.

T6SS effector genes					Cognate T6SS immunity protein genes	
ORF (old locus annotation)	Size (aa)	Bastion6 T6SE (Score)	Target cell	Predicted activity/Domain	ORF/Upstream or downstream^a^	TM or signal peptide/Domain^b^
SED_RS06235 (SeD_A1215)	290	0,711	Prokaryotic	Peptidoglycan hydrolase/LysM	SED_RS06230/Downstream	Signal peptide (Sec/SPI)/Lysozyme inhibitor domain (LprI)
SED_RS06335 (SeD_A1235)	1,406	0,939	Prokaryotic	Peptidoglycan hydrolase/PAAR-RHS-Peptidase_M91	SED_RS06340/Downstream	1 TM/No

a This column indicates if the putative immunity protein gene (ORF) is encoded upstream or downstream the corresponding T6SS effector in a bicistronic unit.

b Presence or absence of transmembrane domains (TM) or a signal peptide and protein domains present in the putative immunity protein genes.

Besides SED_RS06335, our bioinformatic analysis also identified another effector protein encoded within the SPI-19 T6SS gene cluster in *S*. Dublin CT_02021853. This putative effector protein is encoded in SED_RS06235 (Bastion6 score = 0,711). This ORF encodes a 290 amino acid protein with a predicted LysM domain (IPR018392) at its C-terminus. This domain is present in several T6SS effector proteins and has been linked to peptidoglycan hydrolase activity (Fitzsimons et al., 2018), suggesting that SED_RS06235 is a novel T6SS amidase effector protein with antibacterial activity. Indeed, HHPred analysis revealed the presence of a transglycosylase domain in the N-terminal region of SED_RS06235, which is consistent with the notion that this ORF encodes an antibacterial effector protein. Our analysis also revealed that SED_RS06235 is part of a bicistronic unit along with SED_RS06230. This ORF encodes a 146 amino acid protein with a periplasmic-targeting signal peptide. A sequence analysis revealed that SED_RS06230 harbors the LprI PFAM domain (PF07007). This domain is found in LprI, a *Mycobacterium tuberculosis* protein which functions as a lysozyme inhibitor (Sethi et al., 2016). This suggests that SED_RS06235/SED_RS06230 corresponds to a LysM/LysMI E/I pair. Interestingly, the LysM effector of *S*. Enteritidis (SEN1001) has been linked to the ability of the bacteria to colonize the murine host and survive within infected macrophages (Silva et al., 2012). Since SPI-19 has a large internal deletion that inactivated the T6SS_SPI-19_ in *S*. Enteritidis, the mechanism involved in these processes remains unknown.

### The Identified Putative T6SS Effector and Immunity Proteins Are Widely Distributed in *Salmonella* Genomes

The identification of five putative T6SS effector proteins encoded within SPI-6 and SPI-19 T6SS gene clusters of *S*. Dublin CT_02021853 prompted us to investigate the presence and distribution of these proteins in *Salmonella*. To this end, the sequence of each E/I module identified in this study was used in BLASTn searches using publicly available bacterial genome sequences and the distribution of each effector protein was determined (Figures 2B, 3B; Supplementary Table S1).

The analysis of the three putative effector proteins encoded in SPI-6 showed that only a small number of genomes harbors the unannotated E/I module SED_RS24315/SED_RS01915, and they were almost restricted to *S*. Dublin strains (Figure 2B). In contrast, the SED_RS01930/SED_RS01935 E/I module was found in the genome of most *Salmonella* serotypes that harbor SPI-6, including a large number of *S*. Enteritidis strains (33%). Of note, *S*. Enteritidis strains harbor an internal deletion within the SPI-6 T6SS gene cluster that is located upstream of the SED_RS01930/SED_RS01935 E/I module (Blondel et al., 2009).

The two putative effector proteins encoded in SPI-19 that we identified were also widely distributed among *Salmonella* serotypes (Figure 3B). Our analysis showed that the SED_RS06235/SED_RS06230 E/I module is present in the genome of strains of every *Salmonella* serotype harboring SPI-19. However, the SED_RS06335/SED_RS06340 E/I module was found in a larger number of *Salmonella* genomes, including those not previously described to carry SPI-19 (Supplementary Table S1). As in the case of the SED_RS01930/SED_RS01935 E/I pair encoded in SPI-6, a large number of *S*. Enteritidis strains were also found to encode the two T6SS_SPI-19_ effector proteins identified in this study. Although *S*. Enteritidis strains harbor large deletions within SPI-6 and SPI-19 that most likely make the corresponding T6SSs non-functional (Blondel et al., 2009), the presence of multiple E/I modules in these clusters suggests that *S*. Enteritidis retains immunity to many T6SS_SPI-6_ and T6SS_SPI-19_ effector proteins.

### Contribution of Selected E/I Modules to Interbacterial Competition in *S*. Dublin CT_02021853

**Figure 4:**
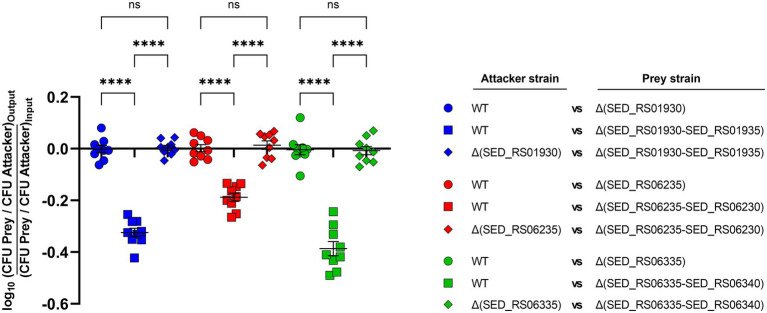
Contribution of selected effectors and effector/immunity protein pairs to interbacterial competition in *S*. Dublin. Bacterial suspensions of attacker and prey strains adjusted to an OD_600nm_ of 0.5 were mixed at a 1:1 ratio. Then, 25 μl of the mixture was incubated at 37°C for 24 h in triplicate on MacConkey agar plates. Bacterial counts recovered from each competition assay were calculated by plating serial 10-fold dilutions on LB agar plates with the appropriate antibiotics (Kan in the case of the attacker strain and Cam in the case of the prey strain). Data show the prey to attacker CFU ratio normalized to the inoculum and expressed as log_10_. Error bars indicate standard error. Statistical significance was determined using a one-way ANOVA followed by Tukey’s multiple comparisons test (^****^p < 0.0001; ns, not significant).

We aimed to evaluate if a selected group of E/I modules identified by our bioinformatic analysis contribute to interbacterial competition in *S*. Dublin. To this end, we chose E/I modules SED_RS01930/SED_RS01935 (located in SPI-6), SED_RS06235/SED_RS06230, and SED_RS06335/SED_RS06340 (both located in SPI-19) and generated non-polar deletion mutants of either the effector-encoding gene or the whole E/I module in *S*. Dublin CT_02021853. Next, we performed interbacterial competition assays using different combinations of attacker and prey strains. Noteworthy, in all three E/I modules analyzed we observed that the survival of the mutant lacking the effector-encoding gene (and keeping intact the gene encoding the cognate immunity protein) was not affected after coincubation with the wild-type strain (Figure 4). These results indicate that each immunity protein is able to counteract the toxic activity of the cognate effector protein delivered by the attacker. In contrast, the survival of the mutant lacking the whole E/I module was reduced after coincubation with the wild-type strain. In fact, single mutants lacking E/I module SED_RS01930/SED_RS01935, SED_RS06235/SED_RS06230, and SED_RS06335/SED_RS06340 were recovered 2-, 1.5-, and 2.4-fold less than the wild-type after coincubation, respectively (Figure 4). Finally, the survival of mutants lacking either the effector-encoding gene or the corresponding E/I module were identical when they were coincubated (Figure 4). These results confirm that modules SED_RS01930/SED_RS01935, SED_RS06235/SED_RS06230, and SED_RS06335/SED_RS06340 contribute to interbacterial competition mediated by either T6SS_SPI-6_ or T6SS_SPI-19_ in *S*. Dublin CT_02021853. Also, these results indicate that SED_RS01930, SED_RS06235, and SED_RS06335 encode functional effector proteins, while SED_RS01935, SED_RS06230, and SED_RS06340 encode their cognate immunity proteins. Further experimental evidence is required to fully confirm that each identified effector protein is secreted *via* T6SS_SPI-6_ and/or T6SS_SPI-19_.

## Conclusions

Altogether, our results indicate that both T6SS_SPI-6_ and T6SS_SPI-19_ contribute to interbacterial competition by *S*. Dublin CT_02021853. We identified novel effector proteins and cognate immunity proteins encoded in SPI-6 and SPI-19 T6SS gene clusters and confirmed the contribution of three novel E/I pairs to interbacterial competition in *S*. Dublin CT_02021853. The biochemical characterization of the putative effector proteins identified is currently undergoing in our laboratory.

The question of why *S*. Dublin acquired and maintained two distinct and functional T6SSs remains unanswered. We and others have proposed that the presence of multiple T6SSs could contribute to *Salmonella* fitness in different environments and/or hosts. Thus, the presence of putative antibacterial T6SS effector proteins suggests that *S*. Dublin may use them to differentially target bacterial cells within the cattle microbiota. Additional studies are required to confirm this hypothesis.

## Data Availability Statement

The original contributions presented in the study are included in the article/Supplementary Material; further inquiries can be directed to the corresponding authors.

## Author Contributions

FA, CB, CS, and DP: conceptualization, formal analysis, validation, writing-original draft preparation, and writing-review and editing. FA, CB, MB-I, DR, CS, and DP: methodology and investigation. CB, CS, AM-S, and DP: resources, project administration, and funding acquisition. CS and DP: supervision. FA, CB, and DP: visualization. All authors read and approved the final manuscript.

## Funding

This work was supported by FONDECYT grant 1181167, ANID Millennium Science Initiative/Millennium Initiative for Collaborative Research on Bacterial Resistance, MICROB-R, NCN17_081. CS was supported by FONDECYT grants 1171844, and 1212075. CB was supported by FONDECYT grant 1201805, ECOS-ANID ECOS200037, and HHMI-Gulbenkian International Research Scholar grant #55008749. FA was supported by CONICYT/ANID fellowship 21191925.

## Conflict of Interest

The authors declare that the research was conducted in the absence of any commercial or financial relationships that could be construed as a potential conflict of interest.

## Publisher’s Note

All claims expressed in this article are solely those of the authors and do not necessarily represent those of their affiliated organizations, or those of the publisher, the editors and the reviewers. Any product that may be evaluated in this article, or claim that may be made by its manufacturer, is not guaranteed or endorsed by the publisher.
