# Supplementary material for: Identification of Type VI Secretion Systems Effector Proteins That Contribute to Interbacterial Competition in Salmonella Dublin
Source: Front Microbiol. 2022 Feb 10;13:811932. doi: 10.3389/fmicb.2022.811932 (PMC8867033; doi:10.3389/fmicb.2022.811932)
Supplement: Supplementary file 1 [file Data_Sheet_1.PDF]

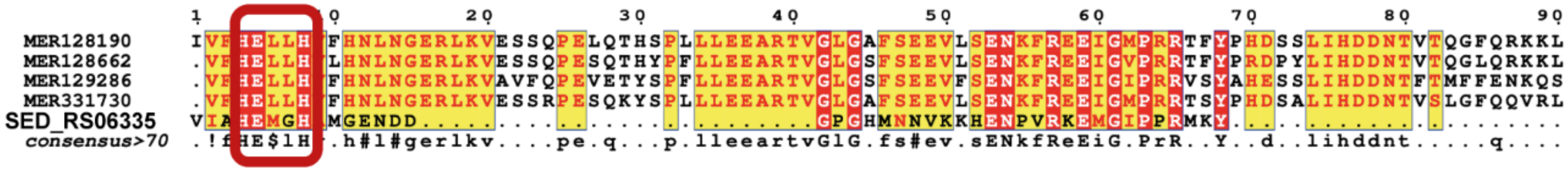

Figure S1. Multiple sequence alignment of the M91 protein domain of SED_RS06335 and metalopeptidases representatives of the M91 family of proteins. BLASTp alignments were performed using T-Coffee Expresso and visualized by ESPript 3. Amino acids with a red background correspond to positions with 100% identity. The HExxH zinc-coordinating and catalytic residues are highlighted.
